# Behcet disease: A case report on the utilization of T1‐weighted black blood fat‐suppressed sequences for the detection of venous vasculitis

**DOI:** 10.1002/ccr3.8479

**Published:** 2024-02-09

**Authors:** Daniel Weiss, Elisabeth Appel, Bernd Turowski

**Affiliations:** ^1^ Department of Diagnostic and Interventional Radiology, Medical Faculty and University Hospital Düsseldorf Heinrich‐Heine‐University Düsseldorf Düsseldorf Germany

**Keywords:** CNS involvement, inflammatory, MRI, vasculitis

## Abstract

T1‐weighted black blood FS sequences may provide a useful addition to imaging protocols in detection of subtle changes in venous vasculitides and, therefore, may have an impact on treatment options.

## Graphical abstract

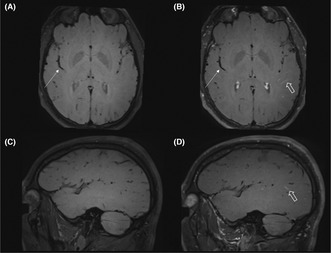

## 1 INTRODUCTION

Behcet disease (BD) is a multisystemic chronic vasculopathy with highest prevalence in the Mediterranean and the Middle East, and it is relatively uncommon among patients of Central European descent. The typical initial symptoms of BD include oral and genital ulcerations, as well as ocular lesions. Furthermore, there are various additional symptoms involving cardiovascular, thoracic, gastrointestinal, musculoskeletal, and central nervous system (CNS) manifestations.^1^ Correctly diagnosing BD can be challenging due to the variability of symptoms, and comprehensive scoring systems have been developed to assess the likelihood of the presence of BD.^2^

Neurological symptoms in the context of BD such as headache or sensory impairments may appear nonspecific and pose a particular challenge, as they can mimic other conditions.^3^ BD patients often report headaches, but these are frequently not clearly attributable to the disease and may also be explained by tension headaches or migraines.^4^ Consequently, headaches are currently not included in the diagnostic criteria for BD. Alongside clinical symptoms and lumbar puncture, magnetic resonance imaging (MRI) plays a decisive role in these cases, as it may demonstrate parenchymal, meningeal or cerebrovascular involvement of BD.

The following case is significant because we present a mild case of BD, in which we employed a rarely used diagnostic approach involving special MRI sequences to support the confirmation of the diagnosis.

## 2 CASE DESCRIPTION

### 2.1 Patient information

A 29‐year‐old male of Central European descent sought evaluation at the neurological clinic of the local university hospital following a referral from his primary care physician. The primary concern was to investigate the suspicion of BD, prompted by recurring holocephalic headaches, as well as persistent oral and genital ulcerations. Notably, the patient also reported substantial neck pain concurrent with the headaches and occasional episodes of dizziness. The patient was known to have a positive HLA‐B51. Further complicating the clinical picture, the patient had a medical history encompassing multiple episodes of aseptic meningitis. Additional pre‐existing conditions included eosinophilic esophagitis, diverticulosis, and adenoid hyperplasia.

### 2.2 Clinical findings

In the context of clinical examination, when there is suspicion of CNS involvement in BD, a neurological examination, rheumatological evaluation, and ophthalmological consultation are particularly crucial. These assessments play an important role in securely identifying the various symptoms associated with the disease.

The comprehensive neurological examination at admission revealed the following findings: The patient demonstrated full consciousness, alertness, and responsiveness, presenting in an affable manner with orientation across all qualities. Notably, no signs of meningism or Lhermitte's sign were observed. Pupils were bilaterally equal, moderately sized, and promptly responsive to light. Visual field assessments proved unremarkable for all quadrants, and visual acuity was within the normal range. Oculomotor function was intact, devoid of any nystagmus. Facial sensation and hearing were both normal, and there was no evidence of dysphagia. A meticulous evaluation of the remaining cranial nerves unveiled no pathological findings. Motor function examinations indicated unremarkable findings, with the patient displaying full strength in all extremities and eliciting brisk, symmetrical reflex responses. Sensation, both in terms of quality and symmetry, was intact. Coordination testing yielded no evidence of dysmetria or ataxia. The gait pattern was normal, and further gait tests produced unremarkable results, collectively indicating an overall unremarkable neurological status.

BD patients frequently describe experiencing emotions such as depression, restlessness, and fatigue. However, it is noteworthy that the psychiatric assessment conducted upon admission yielded unremarkable results.

After a thorough ophthalmological examination, no abnormalities were observed, particularly no signs of BD, such as posterior uveitis. During the rheumatological examination, the patient presented in excellent overall health. Notably, there were no current manifestations of vasculitic or collagenous‐typical alterations in the skin and mucous membranes, and no observable psoriasis on the visible integument. Absence of phlebitis and folliculitis was evident. Despite the presence of numerous large tattoos, there were no discernible signs of heightened skin irritation. The musculoskeletal assessment revealed an age‐appropriate condition, devoid of any active synovitis. The musculature demonstrated bilateral symmetry and normal functionality. No complaints of myalgias or myogeloses were reported, and peripheral edemas were notably absent. The patient's extensive tattooing did not show any indications of causing undue skin irritation.

### 2.3 Diagnostic assessment

In addition to the previously described physical, discipline‐specific examinations, further instrumental investigations are crucial for confirming the diagnosis.

**FIGURE 1:**
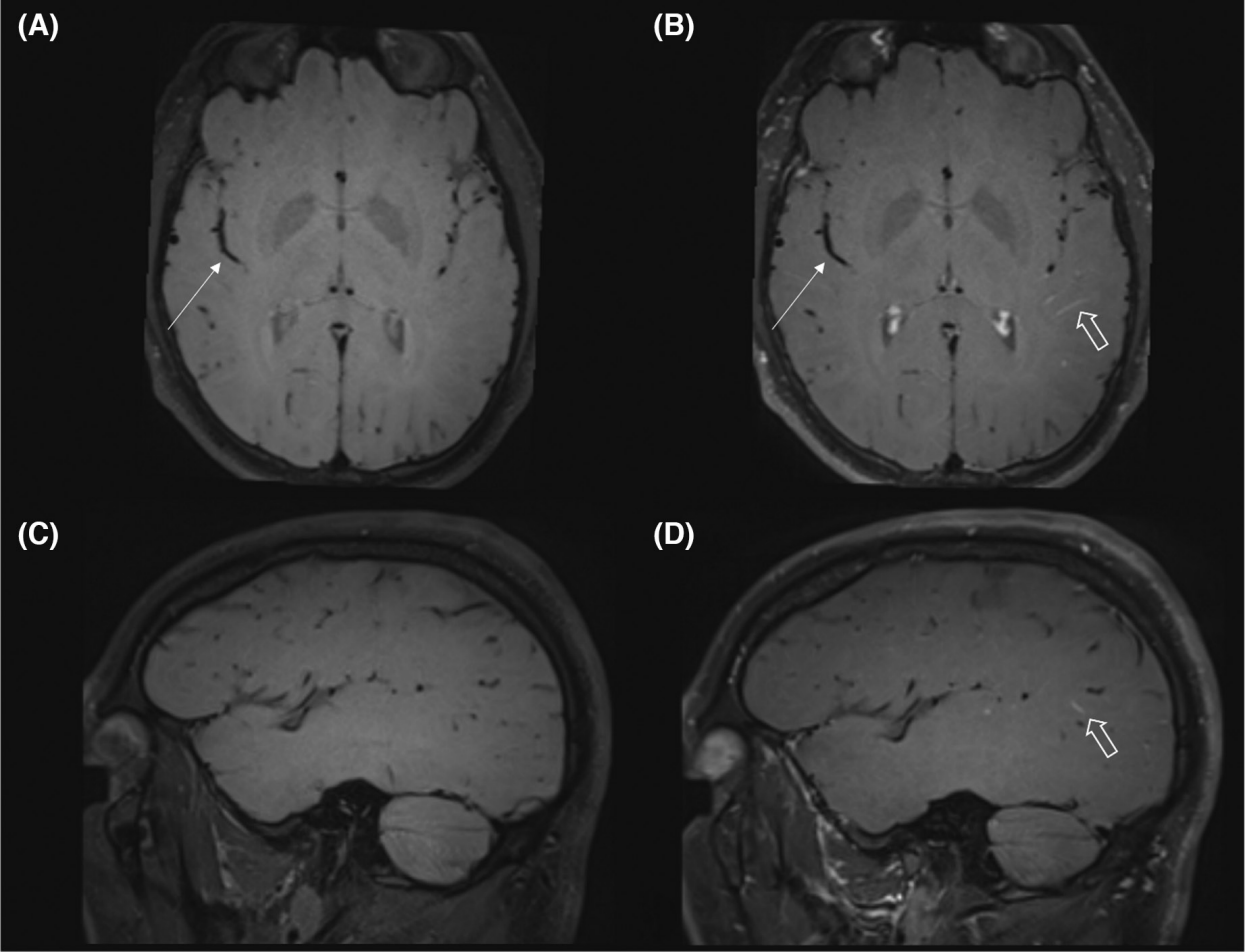
Pathological contrast enhancement of cortical veins in T1‐weighted black blood fat‐suppressed (FS) sequences. (A) axial and (B) sagittal T1‐weighted black blood fat‐suppressed sequences. (C) axial and (D) T1‐weighted black blood FS sequences after contrast agent administration. White arrow: right insular M2‐Segment of the middle cerebral artery without pathological wall enhancement. White open arrow: after contrast agent administration a pathological contrast enhancement of the vessel walls of cortical veins of the left hemisphere is shown.

Cerebral MRI was performed to correlate clinical symptoms with possible cerebrovascular involvement in BD. The MRI protocol (MAGNETOM Skyra, 3 T, Siemens) was adapted accordingly and consisted of coronal T2‐weighted turbo‐spin‐echo (TSE) dark‐fluid sequences (5.5 mm), axial T2‐weighted TSE sequences (5.5 mm), axial susceptibility‐weighted images (1 mm), 3D phase‐contrast MR‐angiography (1 mm), and 3D T1‐weighted black blood fat‐suppressed (FS) sequences with and without contrast agent (Gadolinium, 1.0 mmol/mL, 0.1 mL/kg, TR:862, TE:18, 0.9 mm). The patients' MRI did not show cerebral venous sinus thrombosis, pathological leptomeningeal contrast enhancement or typical parenchymal lesions. However, contrast enhanced T1‐weighted black blood FS sequences showed pathological wall enhancement of cortical veins (Figure 1).

To further investigate the patient's neck pain, an MRI of the cervical and thoracic spine was also conducted. The imaging revealed preserved alignment of the spine without significant degenerative changes. There was no disc herniation or protrusion, and no evidence of neuroforaminal, recessal, or spinal stenosis. There were no indications of inflammatory changes in the spinal cord.

In further neurological examinations, evoked potentials were also derived. Motor evoked potentials, somatosensory evoked potentials, and visually evoked potentials were all within normal limits, indicating unremarkable neurophysiological responses.

Extracranial duplex ultrasound revealed no abnormalities, ruling out hemodynamically relevant stenoses. Intracranial duplex ultrasound findings were similarly unremarkable. The recorded flow profiles exhibited normal characteristics, with a velocity of 100 cm/s in the right and 88 cm/s in the left middle cerebral artery, indicating a well‐maintained cerebrovascular status.

Laboratorially, a positive HLA‐B51 value was present, and all other parameters investigated in the context of vasculitis evaluation (e.g., ANA, c‐ANCA, etc.) including the soluble interleukin‐2 receptor were unremarkable. Additionally, elevated blood lipid levels were observed, including increased LDL, HDL, cholesterol, and triglyceride values. The rest of the laboratory results were within normal ranges.

Despite receiving comprehensive information about the significance of the procedure, the patient opted to decline a lumbar puncture, expressing a fear of the process. As a result, the lumbar puncture, a diagnostic measure deemed valuable in this clinical context, was regrettably not conducted.

The performed pathergy test yielded a positive result. This was conducted in a standardized manner: a 20‐gauge needle puncture, possibly followed by an additional injection of 0.1 mL 0.9% saline solution on the volar aspect of the forearm. The development of an erythematous papule with a diameter greater than 2 mm was found after approximately 24 hours after puncture (obligatory within 48 hours) indicated a positive test result.

According to the International Criteria for BD (ICBD), a score of six points was obtained (genital and oral ulcerations, neurological involvement, and a positive pathergy test).^2^ A diagnosis of BD is possible with a score equal to or greater than four points.

### 2.4 Therapeutic intervention and outcome

Considering the findings, a diagnosis of a mild form of BD was established. This prompted an interdisciplinary discussion of potential therapeutic options. Initially, a 5‐day course of provisional steroid pulse therapy was initiated during the inpatient stay, involving the administration of 250 mg of Methylprednisolone per day. This intervention yielded a modest improvement in the patient's condition. Subsequently, further therapeutic strategies were considered and discussed in collaboration with various medical disciplines.

Fundamentally, there are several therapeutic options, such as azathioprine, certolizumab, or high‐dose steroids to consider. However, due to the symptomatology and the associated side effects of the therapy, a decision was initially made against extensive treatment. Given the mild manifestation of symptoms, an attempt was made with a very low‐dose colchicine therapy. This involved the administration of colchicine, with a dosage of 0.5 mg in the morning and 0.5 mg in the evening.

Additionally, regular monitoring of blood parameters and a follow‐up appointment in 2 months were recommended to evaluate the treatment's success and consider potential adjustments to the colchicine dosage or modifications to the therapy. The patient was discharged in good and unchanged condition, returning to his home environment.

In the follow‐up after 2 months, the patient was symptom‐free and exhibits no therapy‐related side effects, which is why the treatment plan was continued. A follow‐up appointment has been scheduled for 6 months.

## 3 DISCUSSION

In addition to the neurological examination and serological analysis, MRI plays a decisive role in detecting CNS involvement in BD.^5^, ^6^ In this particular case, MRI is even more crucial because the patient declined a lumbar puncture, potentially preventing the acquisition of significant diagnostic clues for BD.^2^ While the patient exhibited a positive pathergy test, it is essential to note that this test may also yield positive results in other conditions, such as neutrophilic dermatoses. Therefore, its interpretation should be considered within the context of the entire diagnostic set.^7^, ^8^ Furthermore, although he exhibited clinical symptoms consistent with a Behcet's diagnosis, these alone were not sufficiently pronounced to permit a reliable clinical diagnosis.

MRI, in general, has proven particularly useful in visualizing parenchymal lesions, which may be present in 60% of all cases with involvement of the CNS. However, cerebrovascular involvement of larger vessels has been described, mostly presenting itself as cerebral venous sinus thrombosis.^4^, ^5^ Nevertheless, imaging of inflammatory involvement of smaller vessels was not possible with standard MRI sequences.

In the context of this issue, the use of T1‐weighted black blood FS sequences is a promising approach. They have gained importance in diagnosis of inflammatory vascular diseases of the CNS. Suppressing the blood flow signal enables visualization of the vessel wall itself. After administration of contrast agent, enhancement of the vessel wall can be depicted in the event of an inflammatory change.^9^ Some studies have already shown potential benefit of T1‐weighted black blood FS sequences in detection of cerebral thrombosis, although larger prospective studies are required.^10^ Nevertheless, there are some pitfalls in interpretation of findings: in proximal vessel segments of intracranial arteries, enhancement may occur due to accompanying vasa vasorum.^11^ Furthermore, arteriosclerotic lesions may also depict an eccentric change of the vessel wall.^12^ However, concentric enhancement in distal vessels is thought to be more likely related to pathological conditions.^11^

In this case, a pathological contrast enhancement of the vessel walls of cortical veins of the left hemisphere is shown (Figure 1). To date, the utilization of T1‐weighted black blood FS sequences in the detection of venous vasculitis has not yet been described. Nevertheless, the combination of symptoms and the enhancement of the cortical veins strongly suggests venous vasculitis in this case. The significance of MRI in this case is particularly grounded in the fact that the remaining symptoms and diagnostic indicators for BD are relatively subtle. However, the patient experienced significant distress, warranting further investigation. To initiate a meaningful therapy, a secure diagnosis is essential, which, in this instance, was complicated by the patient's refusal of a lumbar puncture. While the described symptoms meet the diagnostic criteria for a diagnosis of BD according to ICBD, confirmation of CNS involvement should ideally be obtained through an MRI or lumbar puncture.^2^, ^13^ Nevertheless, larger‐scale studies are required to determine the accuracy of T1‐weighted black blood FS sequence in the diagnosis of venous vasculitis.

## 4 CONCLUSION

Targeted imaging protocols are crucial for accurate diagnosis. In this case, the use of the T1‐weighted black blood fat‐suppressed sequence proved to be a valuable addition to imaging protocols, facilitating identification of subtle changes in venous vasculitides.

## AUTHOR CONTRIBUTIONS

**Daniel Weiss:** Conceptualization; data curation; formal analysis; investigation; methodology; project administration; supervision; validation; visualization; writing – original draft; writing – review and editing. **Elisabeth Appel:** Conceptualization; data curation; investigation; methodology; supervision; validation; writing – review and editing. **Bernd Turowski:** Conceptualization; data curation; formal analysis; investigation; methodology; project administration; supervision; validation; writing – review and editing.

## FUNDING INFORMATION

No funds, grants, or other support were received.

## CONFLICT OF INTEREST STATEMENT

All authors declare that they have no competing interests.

## ETHICS STATEMENT

This case report was conducted in accordance with the Declaration of Helsinki. The Institutional Review Board of our hospital did not require ethics approval in this case report.

## CONSENT

Written informed consent was obtained from the patient to publish this report in accordance with the journal's patient consent policy.

## Data Availability

The data that support the findings of this study are available from the corresponding author upon reasonable request.
